# Associative Account of Self-Cognition: Extended Forward Model and Multi-Layer Structure

**DOI:** 10.3389/fnhum.2013.00535

**Published:** 2013-08-30

**Authors:** Motoaki Sugiura

**Affiliations:** ^1^Institute of Development, Aging and Cancer, Tohoku University, Sendai, Japan; ^2^International Research Institute of Disaster Science, Tohoku University, Sendai, Japan

**Keywords:** self, self-recognition, self-awareness, body-ownership, self-agency, social cognition, associative learning, neuroimaging

## Abstract

The neural correlates of “self” identified by neuroimaging studies differ depending on which aspects of self are addressed. Here, three categories of self are proposed based on neuroimaging findings and an evaluation of the likely underlying cognitive processes. The physical self, representing self-agency of action, body-ownership, and bodily self-recognition, is supported by the sensory and motor association cortices located primarily in the right hemisphere. The interpersonal self, representing the attention or intentions of others directed at the self, is supported by several amodal association cortices in the dorsomedial frontal and lateral posterior cortices. The social self, representing the self as a collection of context-dependent social-values, is supported by the ventral aspect of the medial prefrontal cortex and the posterior cingulate cortex. Despite differences in the underlying cognitive processes and neural substrates, all three categories of self are likely to share the computational characteristics of the forward model, which is underpinned by internal schema or learned associations between one’s behavioral output and the consequential input. Additionally, these three categories exist within a hierarchical layer structure based on developmental processes that updates the schema through the attribution of prediction error. In this account, most of the association cortices critically contribute to some aspect of the self through associative learning while the primary regions involved shift from the lateral to the medial cortices in a sequence from the physical to the interpersonal to the social self.

## Introduction

A sporadic quest for the neural basis of “self” using functional neuroimaging appears to have emerged at the end of the last century. A number of researchers were interested in the cognitive processes related to physical self-awareness during action and consequential sensation (McGuire et al., 1996a; Blakemore et al., 1998; Fink et al., 1999), whereas others investigated the self-relevance of memory and knowledge (Fink et al., 1996; Craik et al., 1999; Kelley et al., 2002). An initial study evaluating stimulus-independent thought (McGuire et al., 1996b) drew attention to the relevance of spontaneous neural activity during a conscious resting state (Raichle et al., 2001) to the self-related cognitive process (Gusnard et al., 2001). The subsequent surge in studies of self-face recognition (Keenan et al., 2000; Kircher et al., 2000; Sugiura et al., 2000) rested primarily on an evolutionary or developmental perspective. Perceptions of others’ communicative intentions toward the self (Kampe et al., 2003) and perspective-taking (Vogeley et al., 2004) appear to be other independent issues. It did not take long for researchers to realize that the cortical regions supporting self-specific or self-relevant activation were far from consistent across studies.

The response of researchers to this chaotic situation has also varied considerably. Some have been pessimistic regarding the existence of a special neural system for the self (Gillihan and Farah, 2005; Platek et al., 2008), whereas others have remained optimistic and attempted to identify such a system by sorting out how previous studies have addressed the concept of self. One such approach highlighted cortical midline structures (Northoff and Bermpohl, 2004; Northoff et al., 2006), another focused on the right lateral cortices (Keenan et al., 2000; Feinberg and Keenan, 2005), and yet another tried to reconcile these two views (Uddin et al., 2007).

This paper attempts to provide a unified framework for the neural underpinnings of the self in the context of a pessimistic stance toward the existence of a self-specific neural system. First, neuroimaging studies investigating self-related processes are reviewed, and the concepts of the self and their related brain networks are roughly categorized into three areas. Then, a unique characteristic or computational architecture that is potentially common to the processes of the three categories is proposed. Furthermore, I propose a layer structure characterized by cross-layer dynamics that operates across these three categories. Finally, the manner in which self is related to midline brain regions in this model is discussed. The proposed model is an updated version of one that was previously presented (Sugiura, 2011).

## Three Categories of Self and Neuroimaging Findings

The concepts of self or self-related processes addressed in previous neuroimaging studies may be divided into three categories: the physical self, the interpersonal self, and the social-value of the self. This categorization takes into account the presumably developmental context in which self-awareness is experienced as well as the distribution of reported self-relevant activation. The known basic functional characteristics of the relevant activated regions will be briefly discussed.

### Physical self

One prominent category of self is the body-grounded self that dissociates one’s physical existence from the external environment. Research on this category often focuses on the ability to dissociate self from non-self, such as one’s own face from another’s face, one’s own body from another’s body, one’s own action from another’s action, and one’s own voice from another’s voice. This category of self is conceptually unambiguous and experimental manipulation is often clear. The concept of self in this category may overlap with that of James’ description of “physical self” (James, 1890) or other researchers’ descriptions of “ecological self” (Neisser, 1988) and “minimal self” (Gallagher, 2000). This category of self is of central interest to psychologists studying animals and infants. There is a strong expectation of the existence of specialized neural regions underpinning self-recognition that stems from the fact that only a few species of animals show evidence of visual self-recognition in the mirror (Gallup, 1982; Suddendorf and Collier-Baker, 2009).

Neuroimaging studies investigating the cortical foundation of the physical self have adopted three major experimental approaches. The first approach is to contrast brain activation during the perception of a visual or auditory stimulus relevant to one’s own body with activation during the perception of perceptually similar but self-irrelevant stimuli. Studies using this approach present subjects with a picture or video clip of a face or body (Kircher et al., 2000; Platek et al., 2004, 2006; Sugiura et al., 2005a, 2006, 2008, 2012; Uddin et al., 2005; Devue et al., 2007; Kaplan et al., 2008; Ferri et al., 2012; Oikawa et al., 2012), or a recorded voice (Nakamura et al., 2001); the required task is either explicit or implicit recognition (e.g., passive viewing or performance of an unrelated task). The second approach addresses the sense of body-ownership or of body-location drift, which is illusorily induced by a synchronous sensory stimulation including tactile stimuli (Ehrsson et al., 2004, 2005; Tsakiris et al., 2007; Ionta et al., 2011). Contrasting the synchronized and desynchronized conditions can isolate the cortical activation related to such a sense. The third approach deals with the sense of self-agency or self-attribution concerning one’s own actions. Studies have identified neural activation in response to modulated visual feedback during hand action or manipulation of a cursor or agent on a computer (Fink et al., 1999; Farrer et al., 2003, 2008; Leube et al., 2003; David et al., 2007; Schnell et al., 2007; Corradi-Dell’Acqua et al., 2008; Spengler et al., 2009; Yomogida et al., 2010), auditory feedback during speech (McGuire et al., 1996a; Hashimoto and Sakai, 2003; Fu et al., 2006), and tactile feedback while tickling oneself (Blakemore et al., 1998). Some studies have manipulated self-agency simply by instruction (Farrer and Frith, 2002; Schnell et al., 2007), whereas others have examined effects of the trial-by-trial fluctuation in subjective awareness in response to the same stimuli (David et al., 2007; Farrer et al., 2008).

**Figure 1:**
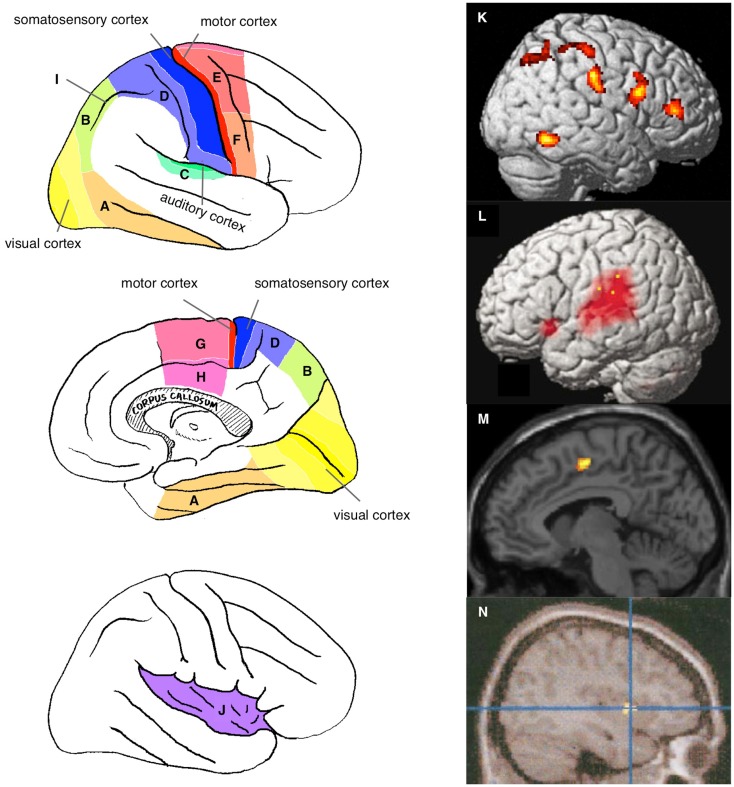
**Neural correlates of the physical self**. Sensory and motor association cortices are schematically illustrated on the lateral (top left panel) and medial (middle left panel) surface of the right hemisphere: visual association cortex [ventral **(A)** and dorsal **(B)** pathways]; auditory association cortex **(C)**; somatosensory association cortex **(D)**; motor association cortices {dorsal **(E)** and ventral **(F)** parts of premotor cortex and medial regions including the supplementary motor area [SMA, **(G)**] and anterior cingulate cortex [ACC, **(H)**]}; and intraparietal sulcus **(I)**. The bottom left panel shows the schema within the opened Sylvian fissure in the right hemisphere to expose the insular cortex **(J)**. Examples of neuroimaging data: activation specifically observed during self-face recognition in picture [**(K)** (Sugiura et al., 2012)]; activation during speech with manipulated auditory feedback of own voice [**(L)** (Hashimoto and Sakai, 2003)]; activation during violated self-agency of control of avatar in computer game [(**M)** (Yomogida et al., 2010)]; and awareness of self-agency of control of cursor in computer game [**(N)** (Farrer and Frith, 2002)].

Although the regions reportedly involved in this activation vary across studies and approaches, they include primarily the sensory and/or motor association cortices (Figure 1) and depend on the sensory modality of the stimulus used. Activation of the visual association cortices, including the ventral and dorsal pathways (Figures 1A,B, respectively), has been reported in studies using visual stimuli to address visual self-face or self-body recognition (Kircher et al., 2000; Sugiura et al., 2005a, 2006, 2008, 2012; Uddin et al., 2005; Platek et al., 2006; Kaplan et al., 2008; Ferri et al., 2012; Oikawa et al., 2012), the illusory sense of body-ownership or location (Ehrsson et al., 2004; Tsakiris et al., 2007; Ionta et al., 2011), and the violation or awareness of action-agency (Fink et al., 1999; David et al., 2007; Corradi-Dell’Acqua et al., 2008; Farrer et al., 2008; Spengler et al., 2009; Yomogida et al., 2010). Auditory association cortices (Figure 1C) are activated during the perception of manipulated feedback of self-voice during speaking aloud (McGuire et al., 1996a; Hashimoto and Sakai, 2003; Fu et al., 2006). Activation of somatosensory association cortices (Figure 1D) has been reported in studies using tactile input to manipulate the agency of self-tickling actions (Blakemore et al., 1998) or to induce an illusory sense of body-ownership or location (Ehrsson et al., 2004; Tsakiris et al., 2007; Ionta et al., 2011).

Activation of motor association cortices is frequently reported in studies in which a subject’s motor action plays a critical role in self-relevance. These regions include the dorsal and ventral aspects of the premotor cortex (Figures 1E,F, respectively) and several medial motor association cortices, such as the supplementary motor area (SMA; Figure 1G) and cingulate motor area (Figure 1H). Examples of such studies include those in which subjects executed motor action while self-agency was manipulated (Farrer and Frith, 2002; Farrer et al., 2003, 2008; David et al., 2007; Schnell et al., 2007; Corradi-Dell’Acqua et al., 2008; Spengler et al., 2009; Yomogida et al., 2010).

However, the involvement of sensory or motor association cortices sometimes has no apparent relevance to the sensory processing of the stimulus or motor output during the task. Such involvement requires explanation in terms of the internal representation of the physical self. For example, activation of somatosensory and premotor cortices has often been reported in studies investigating self-face or self-body recognition using pictures (Uddin et al., 2005; Platek et al., 2006; Sugiura et al., 2006, 2008, 2012; Ferri et al., 2012). The visual–somatosensory association cortex in the intraparietal sulcus (Figure 1I), which has been implicated in the visuospatial motor control of extremities, has been found to be activated in many studies on self when the task is relevant to bodily action either directly (Fink et al., 1999; Ehrsson et al., 2004, 2005; Farrer et al., 2008) or indirectly (e.g., self-face or self-body recognition using pictures involving expressions or actions) (Sugiura et al., 2005a, 2006, 2008, 2012; Oikawa et al., 2012). Additionally, many of these activated areas overlap with regions receiving vestibular input, such as the medial temporal (MT) or medial superior temporal (MST) areas, the ventral intraparietal area (VIP), areas 2v and 3aV, and premotor regions (Smith et al., 2012; zu Eulenburg et al., 2012). The insula (Figure 1J), known to include primary and association cortices for interoception (sense of the physiological conditions of the entire body) and to be involved in a wide range of subjective feelings (Craig, 2002, 2009), is also activated without the manipulation of interoceptive input. Activation of this region was observed during the recognition of self-face or self-body in a picture (Kircher et al., 2000; Devue et al., 2007; Ferri et al., 2012), the sense of action self-agency (Farrer and Frith, 2002; Farrer et al., 2003; Leube et al., 2003; David et al., 2007; Corradi-Dell’Acqua et al., 2008), and the sense of body-ownership (Ehrsson et al., 2004; Tsakiris et al., 2007). These findings may be explained by the fact that bodily self-recognition is grounded by the experience of bodily action accompanied by visual, somatosensory, vestibular, and interoceptive feedback. These interpretations in terms of the representational role of the sensory and motor association cortices will be detailed in Section “Physical Self and Sensorimotor Schema.”

It is interesting to note that some of these parietal sensory association and frontal premotor cortices coincide with the visual–motor association system known as the mirror neuron system (MNS). This apparently contradicts the incompatible concept of “self-specific” and the MNS; which is resolved in the proposed model (See Physical Self and Sensorimotor Schema). Mirror neurons are a class of neurons that have been observed to discharge when a monkey performs a goal-directed motor act as well as when a monkey observes another individual performing the same or a similar motor act (Rizzolatti et al., 2001; Nelissen et al., 2011). In humans, the MNS has been identified as a homolog of the frontoparietal network of mirror neurons in monkeys and is considered to play a critical role in action understanding, imitation, and communication (Rizzolatti and Craighero, 2004; Iacoboni, 2005). Therefore, this system is primarily conceptualized as a mechanism involved in recognizing and interacting with others.

Moreover, these studies reported the activation of several amodal association cortices. Activation of the right lateral prefrontal cortex, specifically the inferior and middle frontal gyri, has often been reported during the recognition of self-face or self-body (Figure 1K) (Platek et al., 2004, 2006; Sugiura et al., 2005a, 2006, 2008, 2012; Uddin et al., 2005; Devue et al., 2007; Kaplan et al., 2008), voice (Nakamura et al., 2001), action-agency violation (Figure 1L) (Fink et al., 1999; Hashimoto and Sakai, 2003; David et al., 2007; Schnell et al., 2007; Farrer et al., 2008), and body-ownership (Ehrsson et al., 2004, 2005; Tsakiris et al., 2007). The manipulation of sensory-feedback for action often activates the temporoparietal junction (TPJ), the posterior part of the superior temporal sulcus (pSTS), and the medial prefrontal cortex (MPFC) (McGuire et al., 1996a; Farrer and Frith, 2002; Farrer et al., 2003, 2008; Hashimoto and Sakai, 2003; Leube et al., 2003; Fu et al., 2006; Spengler et al., 2009; Yomogida et al., 2010), which are typically considered multimodal or amodal association cortices that are implicated in conceptual rather than perceptual processes. These findings will be discussed separately from sensory or motor association cortices in Section “Multi-Layer Structure and Cross-Layer Dynamics.”

### Interpersonal self

When an individual notices that he or she is being looked at or hears his/her own name being called, he/she becomes aware that the attention or intentionality of another person is directed at him/her. This awareness is a basic mindset during social interaction. This aspect of self is obviously distinct from the physical self because it inherently requires the existence of another person. An influential inventory, the Self-Consciousness Scale (Fenigstein et al., 1975), particularly its public subscale, has been developed to measure the degree to which an individual has this type of awareness.

**Figure 2:**
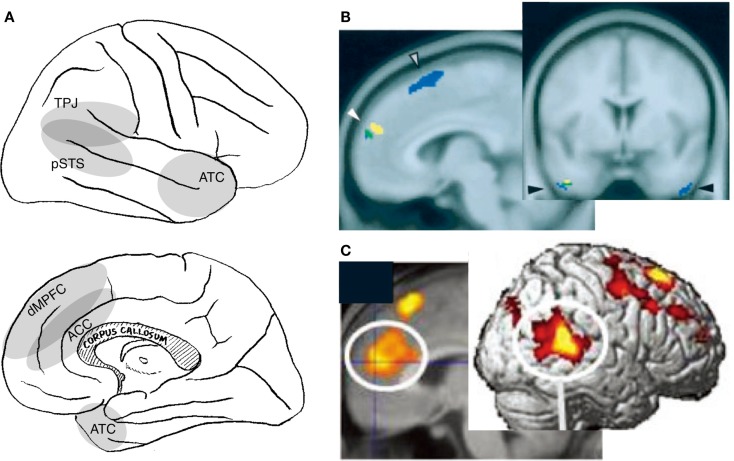
**Neural correlates of interpersonal self**. Relevant cortical areas are schematically illustrated on the lateral (top panel) and medial (bottom panel) surface of the right hemisphere **(A)**. TPJ: temporoparietal junction, pSTS: posterior part of the superior temporal sulcus, ATC: anterior temporal cortex, dMPFC: dorsal part of the medial prefrontal cortex, and ACC: anterior cingulate cortex. Examples of neuroimaging data: activation during the perception of self-directed eye-gaze or the hearing of one’s own name [**(B)**; (Kampe et al., 2003)], and activation during real communication relative to non-real condition [**(C)**; (Jeong et al., 2011)].

The activation related to this awareness is observed in several amodal association cortices in the medial frontal and lateral posterior cortices (Figure 2A). Although varying widely across studies, activation has been identified in the MPFC encompassing the adjacent anterior cingulate cortex (ACC) (Kampe et al., 2003; Schilbach et al., 2006; Steuwe et al., 2012), the TPJ/pSTS (Pelphrey et al., 2004a; Schilbach et al., 2006; Steuwe et al., 2012), the anterior temporal cortex (ATC) (Kawashima et al., 1999; Calder et al., 2002; Kampe et al., 2003; Wicker et al., 2003), the insula (Kawashima et al., 1999; Calder et al., 2002; Schilbach et al., 2006), and the cerebellum (George et al., 2001; Wicker et al., 2003; Schilbach et al., 2006) during the perception of directed, rather than averted, eye-gaze. Activation of the MPFC/ACC, TPJ/pSTS, and ATC has also been reported in studies that compare activation during the hearing of one’s own name with the hearing of others’ names (Figure 2B) (Kampe et al., 2003; Perrin et al., 2005; Tacikowski et al., 2011). Activation is also observed in these regions when subjects believe that they are interacting with a real person rather than engaging in a similar but non-real interaction (Figure 2C) (Rilling et al., 2004; Jeong et al., 2011). Additionally, subjects who score higher on the Self-Consciousness Scale (Fenigstein et al., 1975) show a larger degree of activation in the dorsal part of the MPFC (dMPFC) during a simple sensorimotor (deviant letter detection) task (Eisenberger et al., 2005).

However, previous studies have rarely treated self-awareness as a central concept related to the interpretation of activation in these cortical regions. These regions have often been recognized as a cortical network supporting the inference of another’s mental state, namely, mentalizing or theory of mind (ToM) (Gallagher and Frith, 2003; Frith and Frith, 2006; Senju and Johnson, 2009; Spreng et al., 2009). Furthermore, it has been proposed that this network plays a role in the development of event schemata in general, including person-schema and self-schema (Krueger et al., 2009). The assumed properties of this network provide the basic reasoning for labeling this category of self the “interpersonal self,” which will be detailed in Section “Interpersonal Self and Interpersonal Schema.”

It is worth noting that some of these regions are deactivated rather than activated during self-face recognition. Activation in the TPJ/pSTS or its surrounding cortices is decreased while viewing the self-face compared with familiar or unfamiliar faces (Sugiura et al., 2005a, 2008; Uddin et al., 2005; Devue et al., 2007; Morita et al., 2008), which indicates a clear neural dissociation between physical and interpersonal selves.

### Social-value of self

Self-reflection typically includes thoughts about one’s social-value such as “Am I good-natured?” or “Am I good-looking?” or “Am I intelligent?” or “Am I successful in my career?” Most of the attributes assigned to the self carry some social-value, and individuals are typically aware of the gap between one’s current self and one’s ideal self (Festinger, 1954; Higgins, 1987). This type of social-value is an important aspect of the “social self” according to James (1890) and is assumed to be an important determinant of human behavior [e.g., Self-Efficacy Theory (Bandura, 1982)]. To experimentally address this type of self in neuroimaging studies, self-trait (e.g., personality trait, ability) judgment tasks are typically utilized. Additionally, the perception of the evaluation of self by others, even the perception of others who have a high or low level of an attribute that is significant to the self (Gutierres et al., 1999), is known to affect self-value. Although this type of self resembles the interpersonal self in that it is highly relevant to the existence of another person, the “person” is typically generalized to people or society rather than confined to a specific person. Furthermore, the interpersonal self does not necessarily involve social-value. It is therefore reasonable to categorize self-value separately from interpersonal self.

**Figure 3:**
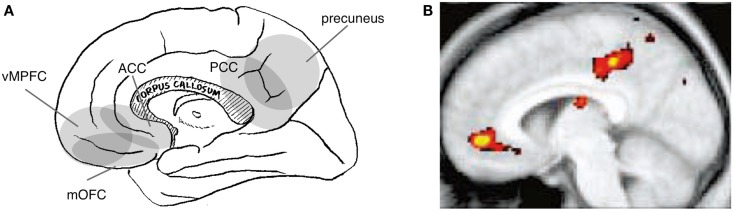
**Neural correlates of the social-value of self**. Relevant cortical areas are schematically illustrated on the medial surface of the right hemisphere. vMPFC: ventral part of the medial prefrontal cortex, ACC: anterior cingulate cortex, mOFC: medial orbitofrontal cortex, and PCC: posterior cingulate cortex **(A)**. An example of neuroimaging data: activation during self-trait judgment about the personality trait adjective [**(B)**; (Kelley et al., 2002)].

Indeed, the cortical regions implicated in self-value have, at least in part, a different distribution than do those implicated in the interpersonal self. Specifically, tasks that are assumed to manipulate the social-value of self typically activate the ventral part of the MPFC (vMPFC) and the posterior part of the cingulate cortex (PCC) or its adjacent medial parietal cortex (i.e., the precuneus) (Figure 3A). Activation of these regions has been reported during self-trait judgment, specifically when contrasted with trait-valence judgment (Craik et al., 1999; Schmitz et al., 2004) or other trait judgments (Figure 3B) (Craik et al., 1999; Kelley et al., 2002; Heatherton et al., 2006; D’Argembeau et al., 2007). Similarly, activation in these regions has been identified when contrasting self-descriptive and non-descriptive trait adjectives (Kircher et al., 2002; Macrae et al., 2004) and when the trait adjective is correlated with self-descriptiveness (Moran et al., 2006). Moreover, the perception of the evaluation of self by others activates these regions (Izuma et al., 2008), particularly in subjects whose self-evaluation is vulnerable to evaluation by others (Somerville et al., 2010). Interestingly, the perception of the evaluation of self by familiar others activates the dMPFC (Korn et al., 2012), which is thought to be the neural correlate of the interpersonal self, rather than vMPFC. This may be explained by the fact that this experimental manipulation affects mental representations of the self in relation to specific others rather than those related to the value of the self, illustrating the conceptual difference between the interpersonal self and the social-value of the self.

Like the physical self, the social self dissociates self and other, but it does so in a different way. The social self encompasses any people or objects that are relevant or behaviorally significant to the self, which are considered “other” in terms of the physical self. The vMPFC and PCC are activated during name and face recognition of oneself and friends relative to recognition of unfamiliar people (Sugiura et al., 2008; Tacikowski et al., 2012), and the vMPFC is correlated with the amount of self-referential thought (D’Argembeau et al., 2005). On the other hand, activation of these regions is often absent when self-trait judgment is compared with trait judgment about familiar people, such as friends and relatives (Schmitz et al., 2004; Benoit et al., 2010). Self-face recognition involves activation of the vMPFC when the number of other faces in the other trials in the task sequence is increased, probably due to the self-value processing induced by social comparison (Sugiura et al., 2012). In a similar task design using young female subjects, activation of the PCC for self-face was enhanced when the female faces in other trials were less attractive, particularly when the subject’s self-esteem was high (Oikawa et al., 2012).

Again, these cortical regions are unlikely to be utilized exclusively for the processing of social-value. The vMPFC and the medial orbitofrontal cortex (mOFC), which is sometimes regarded as identical with or adjacently distinct from the vMPFC, are known to represent the value of objects in general and to play critical roles in value-based decision making (Rangel et al., 2008; Rushworth et al., 2011). This general region comprises a reward system that operates in conjunction with other deep structures, such as the striatum and the midbrain dopamine system, which are sometimes activated during self-trait judgment (Kircher et al., 2002; Moran et al., 2006; Benoit et al., 2010) or perception of self-evaluation by others (Izuma et al., 2008; Korn et al., 2012). The relationship between the reward system and self-related processes is a matter of recent discussion (Northoff and Hayes, 2011). The PCC and the adjacent precuneus are involved in a wide range of highly integrated processes, such as visuospatial imagery, episodic-memory retrieval, and self-referential processes (Wagner et al., 2005; Cavanna and Trimble, 2006). This set of midline cortical regions is also considered to be a major component of the default mode network that is active during a conscious resting state and deactivated during the execution of attention-demanding tasks (Gusnard et al., 2001; Raichle et al., 2001).

### Other aspects of self

One may consider memory, especially autobiographical memory, as a critical factor of self. Numerous functional imaging studies have investigated the neural activity specifically observed during the retrieval of an autobiographical memory. The medial prefrontal and parietal cortices, including the cingulate cortex and the lateral temporal and parietal regions with some regions lateralized to the right (Svoboda et al., 2006; Buckner and Carroll, 2007; Spreng et al., 2009) exhibit activation during such a task. These areas overlap with the cortical regions proposed to be the neural underpinnings of the three categories of self. This suggests that autobiographical memory is not merely a single essential factor but rather the “all-star” of self-related cognitive processes.

Some researchers assume perspective-taking to be a key concept in the distinction of self from other. However, the findings of neuroimaging studies investigating this issue may also be explained by the framework of the three categories of self, particularly the physical and interpersonal selves. Many neuroimaging studies compare first-person (1P) and third-person (3P) perspectives to address this issue. Cortical activation is typically more prominent in a 3P rather than a 1P perspective, but activation of associated brain regions varies widely across studies. These findings were somewhat clarified when perspective-taking was divided into visuospatial and mental perspectives, and activation was assumed to reflect the increased cognitive load related to the non-canonical nature of the 3P perspective. Greater activation for 3P visuospatial perspective-taking relative to 1P visuospatial perspective-taking is typically reported in the visual association and premotor cortices (Vogeley et al., 2004; David et al., 2006, 2008a), which overlap with the neural correlates of the physical self. This finding may be explained by the cognitive load involved in the imaginary physical-location change (i.e., moving self-body) required to obtain a non-canonical 3P viewpoint. Regarding mental perspective-taking, a greater activation for the 3P relative to the 1P perspective is frequently reported in the pSTS/TPJ and dMPFC (Ruby and Decety, 2001, 2003, 2004; David et al., 2008a; Schnell et al., 2011; Ramsey et al., 2013), which overlap with the neural correlates of the interpersonal self. This overlap appears to be reasonable because taking the mental perspective of others (intention, emotion, belief) is synonymous with ToM. On the other hand, many of these studies have reported a greater activation in the 1P compared with the 3P perspective in the MPFC and PCC, which are the proposed neural correlates of the social self. It is often difficult to conclusively attribute this finding to self-cognition, since it is usually explained by either behavioral significance (i.e., the social self) or differential default mode activity (Gusnard et al., 2001; Raichle et al., 2001) due to differences in task difficulty (McKiernan et al., 2003).

## Hypothesis: Three Layers of Internal Schema

In the preceding section, the self was divided into three individual categories that differ according to the related supporting cognitive processes and neural substrates. The current section, however, proposes a common characteristic or computational architecture that underlies the processes of these three categories. In short, the common characteristic is a forward prediction model, which is a rather common and classical conceptualization in models of the physical self. It has been assumed that the physical self is the product of an associative learning process based on the repeated experiences of bodily motion and sensory-feedback.

**Figure 4:**
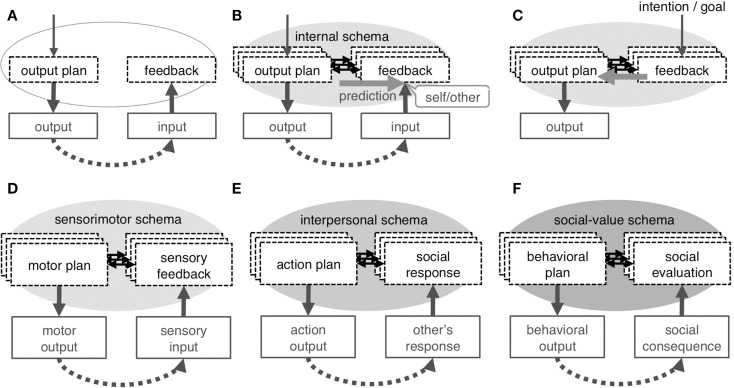
**Concept of internal schema**. Repeated experience of output and feedback input **(A)** results in development of internal schema, which is an association between the neural-representation of an output plan and that of feedback input **(B)**; the schema enables forward prediction, which underlies the sense of self in any category. The schema is not exclusively dedicated to self-cognition but is used as an inverse model to plan output to obtain intended feedback input **(C)**. A different internal schema underlies each category of self: the sensorimotor schema associates motor plan with sensory-feedback to develop the physical self **(D)**, the interpersonal schema associates one’s own action plan with feedback on the social responses by others to develop the interpersonal self **(E)**, and the social-value schema associates one’s behavioral plan with consequential social evaluation to represents the self as a collection of context-dependent social-values **(F)**.

Here, a novel attempt will be made to adapt the forward prediction model to the interpersonal and social selves with the intention of explaining all categories of self within the framework of associative learning. A critical component of this adaptation is the internal schema that denotes the association between the neural-representation of the output plan and the feedback input (Figures 4A,B); this schema is assumed to exist for each target of the output and is modified depending on context. In this view, the self may be defined as a label for the capability of forward prediction (Figure 4B) in any system that has such characteristics. Neuroimaging findings appear to be explained by top-down and bottom-up attention to the schema that is typically driven by task requirements and prediction error, respectively.

Conceptually, the schema is the basis for all cognitive operations, including perception and behavioral control. The schema is used as an inverse model to plan output (Figure 4C) or even to represent each element of the external environment and may be used to simulate a schema of the mind of another; that is, to infer the internal process of others based on the observed output of that person. Given the diverse utility of the schema, the self is only a phenomenon that is occasionally experienced during its functioning, while reflecting the very basic characteristics of the schema. Additionally, a hierarchical layer structure of the three categories of self and the dynamics across the layers are also important features of the proposed model. The hierarchical structure stems from the developmental relationship between the three schemata and serves as the basis of cross-layer interaction, which may be critical for the integrity of the three self-concepts.

### Physical self and sensorimotor schema

The concept of the forward model was first applied to explain the sense of self-agency in action. The sense of self-agency, or the self-attribution of action, is widely assumed to have been derived from the consistency between the sensory input that results from action and the prediction emerging from the action intention or collateral output from the motor system (Figure 4B) (Wegner and Wheatley, 1999; Sato and Yasuda, 2005; David et al., 2008b). One is convinced that the observed action of one’s own hand is actually performed by oneself because the action is somatically experienced (i.e., somatosensory perception) and looks (i.e., visual perception) as predicted. Frith and colleagues (Frith et al., 2000; Frith, 2005) incorporated this conceptualization into a detailed cognitive model by extending the model of feed-forward motor control (Wolpert et al., 1995) to explain the impairment in the sense of self-agency, or the delusion of control, which is a characteristic symptom of schizophrenia. In this model, the prediction of sensory input as a consequence of action is based on intended motor commands and cancels actual input. Subjectively, a successful cancelation is experienced as one’s unawareness of the sensory consequences of one’s own actions and is exemplified as the attenuated sensation of self-generated tickling (Weiskrantz et al., 1971; Blakemore et al., 1999). In functional neuroimaging, this cancelation is detected as an attenuation of the activation related to sensory processing when sensory input is caused by self-generated action rather than being externally produced (Blakemore et al., 1998). More frequently, in fact, this functioning is captured as an increase in activation during the violation of action self-agency due to experimental manipulation (McGuire et al., 1996a; Fink et al., 1999; Hashimoto and Sakai, 2003; Fu et al., 2006; David et al., 2007; Corradi-Dell’Acqua et al., 2008; Farrer et al., 2008; Spengler et al., 2009; Yomogida et al., 2010).

Here, the concept of a sensorimotor schema, or the learned association between one’s motor plan and the feedback sensory input (Figure 4D), is introduced. The sensorimotor schema exists for each effector or movement coordinated by multiple muscles and is adaptively modified depending on physical context, including posture and the external physical environment. The evidence that the schema is indeed constructed through associative learning has been experimentally provided; the repeated experience of an action and its effect on an object on a computer monitor later produce the sense of action self-agency for that virtual effector, and the related neural responses are similar to those seen in previous studies of action self-agency (Schnell et al., 2007; Spengler et al., 2009; Yomogida et al., 2010). The sensorimotor schema allows for the generalization of the forward model to different phenomena of the physical self, such as the sense of body-ownership and self-face recognition in non-contingent images (e.g., static images, prerecorded videos). It has been shown that body-ownership requires a pre-existing internal representation of the position of the limbs (Tsakiris and Haggard, 2005; Costantini and Haggard, 2007). Given that such a representation is constructed and continuously updated by matching the feed-forward prediction and re-afferent sensory input during active movement (Synofzik et al., 2006; Tsakiris et al., 2006), this representation is closely related to or identical with a sensorimotor schema. Self-face recognition ability in a non-contingent image is also likely to depend on a sensorimotor schema. Infants seem to consolidate the visual representation of one’s own face into long-term memory during the experience of viewing a contingent self-face in the mirror; this idea is supported by the observation that self-face recognition first develops in a contingent and then in a non-contingent image (Bigelow, 1981). Therefore, the unique characteristic of the visual representation of the self-face seems to be realized by its association with the experience of action self-agency or body-ownership and, thus, with a sensorimotor schema.

The activation of sensory and motor association cortices related to the physical self is parsimoniously explained by attention to the sensorimotor schema. In this situation, it is advantageous to separate top-down from bottom-up attention. Top-down attention is induced by a variety of experimental manipulations in which the use of information in the schema is necessary or advantageous. Activation of sensory or motor association cortices is observed when perceived motion is explicitly required to be self-produced (Farrer and Frith, 2002) or when the task demands monitoring of one’s own motor action control (Ogawa and Inui, 2007; Schnell et al., 2007). On the other hand, bottom-up attention is typically driven by prediction error, which may contribute to a recalibration of the schema. This line of interpretation most likely refers to neural activation in response to manipulated sensory-feedback, that is, a violation of self-agency in action (McGuire et al., 1996a; Blakemore et al., 1998; Fink et al., 1999; Farrer et al., 2003, 2008; Hashimoto and Sakai, 2003; Fu et al., 2006; David et al., 2007; Schnell et al., 2007; Corradi-Dell’Acqua et al., 2008; Spengler et al., 2009; Yomogida et al., 2010). Activation while experiencing a sense of body-ownership (Ehrsson et al., 2004, 2005; Tsakiris et al., 2007; Ionta et al., 2011) and bodily self-recognition in a non-contingent image (Uddin et al., 2005; Platek et al., 2006; Sugiura et al., 2006, 2008, 2012) may be attributed to either type of attention. The commonality of attention and consciousness may explain the activation of these regions in terms of top-down access to information in the schema. The activation is also attributable to prediction error when perceptual input differs from what was expected: illusion-induced body-ownership may be imperfect, and some strange feelings may remain if the presented self-face picture is somewhat different from what one usually sees in the mirror while one remains sure that the face is one’s own.

The sensorimotor schema explains not only self-cognition but also any cognitive operation related to one’s physical interaction with the external environment. In fact, the forward model, as adapted to self-cognition, was originally developed for motor control (Wolpert et al., 1995), and the concept of sensorimotor schema was adopted from that model. The sensorimotor schema, or the association of the motor plan with sensory-feedback, may be used as an inverse model to calculate the motor plan to obtain the intended sensory-feedback (Figure 4C). This idea is consistent with the conceptual framework of ideomotor theory, which assumes a common coding of action and consequential perception (Prinz, 1997). Furthermore, the sensorimotor schema may play a critical role in an individual’s mental representation of the physical environment. A person can have intention and a motor plan for interaction with many objects in the immediate external environment (e.g., gazing, reaching), and an essential property of the physical environment is this potential interaction, which may be represented in the sensorimotor schema. This notion is compatible with the fact that the cortical areas implicated in sensory or spatial attention overlap primarily with those supporting the sensorimotor schema (Downar et al., 2000; Corbetta and Shulman, 2002).

Furthermore, the sensorimotor schema seems to be exploited to simulate the schema of others; that is, it can be used to infer the intention or action goals of others. The schema may gain the simulation ability by associating one’s own motor output with the perceived contingent motor action of others in an interactive situation where the self and others share an intention or action goal. Such an interactive environment is common in the daily relationship between infants and their caretakers (Kaye and Fogel, 1980; Cohn and Tronick, 1988). This view is consonant with the hypothesis that the MNS is forged by sensorimotor association learning (Heyes, 2010). Further, this view implicate that the mirror neurons are a subcomponent of the simulation-capable sensorimotor schema that associates one’s own motor actions not only with the same action but also with different but related actions of others. This is supported by the fact that, in the cortical areas reported to accommodate mirror neurons in primates, there are a greater number of “counter-mirror neurons,” which code other’s actions that are different from, but related to, one’s own actions (Gallese et al., 1996). Also, in a human neuroimaging study, activation of such regions was greater during observation of other’s actions that were complimentary (i.e., in joint action) to one’s own actions than during observation of immitative actions (Newman-Norlund et al., 2007). Additionally, activation during the observation of another’s actions is not limited to the classic human MNS (i.e., inferior parietal and frontal cortices) but has also been identified in multiple visual and motor association cortices (Caspers et al., 2010).

### Interpersonal self and interpersonal schema

The existence of forward prediction during an individual’s social interaction may be empirically or intuitively plausible. An implicit expectation about the range of possible responses usually arises in situations in which one individual greets another. This is why people become surprised at an unexpected response or the lack of a response from the other person. The range of expected responses greatly differs depending on the identity of the responder (i.e., degree of familiarity and various demographic factors such as age, gender, cultural background, and situational context). The range of expected responses is likely to be updated after repeated experiences of prediction error with a specific familiar person or a specific type of unfamiliar persons.

In this context, it appears reasonable to assume the existence of an interpersonal schema that represents a link between one’s social action (i.e., output plan) and the expected responses (i.e., feedback) of others (Figure 4E). The schema is constructed following repeated exploratory social interactions in daily life; that is, through associative learning involving one’s own social actions toward a person and the feedback (Figures 4A,B). The schema exists for each familiar person or for a specific type of people and is adaptively modified depending on social contextual cues such as time, place, and occasion. These characteristics are comparable to those of the sensorimotor schema in terms of the way the schema develops, that the schema exists for each target of output, and that it is modified depending on context.

Several neuroimaging findings support the conceptualization of the interpersonal schema as the basis of the interpersonal self. Activation related to the interpersonal self appears to be explained by either top-down or bottom-up attention related to the interpersonal schema in a way that is similar to the relationship between the physical self and the sensorimotor schema. It appears reasonable to regard awareness that another’s attention or intention is directed at oneself as an example of top-down attention to the interpersonal schema. In other words, activation of several medial and lateral posterior cortices during the perception of self-directed eye-gaze (Calder et al., 2002; Kampe et al., 2003; Wicker et al., 2003; Pelphrey et al., 2004a; Schilbach et al., 2006; Steuwe et al., 2012), hearing one’s own name being called (Kampe et al., 2003; Perrin et al., 2005; Tacikowski et al., 2011), or real-time interaction with others (Rilling et al., 2004; Jeong et al., 2011) may reflect one’s top-down attention to the representation of the other’s potential response to one’s own social action. Bottom-up attention is also represented by neural activation in this region in response to prediction error or the perception of an unexpected social response as feedback to one’s own action. During a simple two-player strategy game, when the subject believes that the opponent is responding based on the prediction of the subject’s next action, the prediction error of the perceived opponent’s action induces activation in these regions (Hampton et al., 2008).

Furthermore, the functioning of the interpersonal schema is not specific to self-cognition but also relates to any cognitive operation associated with interpersonal interaction. The schema may be used as an inverse model to calculate the behavioral plan of social action toward another person to obtain an intended social response (Figure 4C). Accordingly, the neural correlates of the schema are more activated during speech production toward a virtual agent than during an overt description of the situation (Sassa et al., 2007). An individual can have an intention and a plan of social interaction (or of no interaction) in relation to many people in the immediate social environment. An essential property of the immediate social environment is this potential, which is represented in the interpersonal schema. A similar notion, referred to as “social attention,” is thought to be supported by the same cortical network (Nummenmaa and Calder, 2009). Furthermore, the simulation capacity of the interpersonal schema, or the inference of the intention and plan of another’s social action based on perceived action, may partially overlap with ToM and may explain the overlap of their neural correlates (Gallagher and Frith, 2003; Frith and Frith, 2006; Spreng et al., 2009). However, it is important to note that ToM addresses both the social and non-social beliefs and intentions of others. According to a theory of the role of this network in the development of event schemata in general, the MPFC is assumed to support an abstract dynamic summary representation in the form of event simulators, and its interaction with posterior cortical areas is assumed to comprise knowledge of social events (Krueger et al., 2009). Activation of the implicated cortical regions has been reported in studies evaluating the detection of prediction-violating behavior or objects in the absence of self-involvement or social context (Grezes et al., 2004; Pelphrey et al., 2004b; Wakusawa et al., 2009). Thus, the mature interpersonal schema functions independent of self-cognition and comprises one aspect of a cognitive system supporting higher social and non-social processes. Nevertheless, working from the perspective that the evolution of intelligence in primates has been driven by social demand (Humphrey, 1976; Byrne and Whiten, 1988), it is tempting to assume that the initial interpersonal schema is the origin of the entire system.

### Social-value schema

Forward prediction is also plausible during the evaluation of one’s own social-value. People are surprised when they receive an extremely high or low evaluation for a certain behavior; that is, an individual is relatively unaware of having obtained an evaluation when the evaluation is within the predicted range. It is assumed that humans have multiple self-concepts and that self-value is dependent on social role (e.g., parent, friend, worker) (Stryker and Statham, 1985; Markus and Cross, 1990; Roberts and Donahue, 1994). Thus, it appears reasonable to assume a specific range of the predicted evaluation for each contextual role, and this is updated through the feedback of prediction error.

It is assumed herein that the social-value schema represents a link between one’s social behavior (i.e., output plan) and the predicted evaluation of this behavior (i.e., feedback) (Figure 4F). This schema is constructed for each contextual role through repeated experiences with social evaluations, which result in the learning of associations between one’s own social behaviors and the evaluative feedback they elicit (Figures 4A,B). Again, these characteristics are comparable to those of the sensorimotor or interpersonal schemas in terms of the way the schema develops, that the schema exists for each target of output, and that it is modified depending on context. This idea is congruent with the known general roles of the neural correlates of this schema: the vMPFC (as well as the ACC and mOFC) represents values (Rangel et al., 2008; Rushworth et al., 2011), and the PCC (and precuneus) processes the different aspects of social or autobiographical contexts (Addis et al., 2004; Gilboa et al., 2004; Chiao et al., 2009) and different types of perspectives (Vollm et al., 2006; Mano et al., 2009).

Neuroimaging findings relevant to the social-value of the self are likely explained by either top-down or bottom-up attention related to the social-value schema. The activation of the vMPFC and PCC during self-trait judgment (Craik et al., 1999; Kelley et al., 2002; Schmitz et al., 2004; Heatherton et al., 2006; D’Argembeau et al., 2007), perception of self-descriptive trait adjectives (Kircher et al., 2002; Macrae et al., 2004; Moran et al., 2006), and perception of self-evaluation by others (Izuma et al., 2008; Somerville et al., 2010) may reflect top-down attention to the social-value schema. Activation of these regions in terms of bottom-up attention in response to unexpected evaluations of one’s behavior was found in a study using monetary rewards for a simple estimation game involving a pair of players. These regions exhibited greater activation when the payment to the two players was unequal for the same correct performance (i.e., prediction error in evaluation) than when it was equal (Fliessbach et al., 2007).

The functioning of the social-value schema is also not specific to self-evaluation but operates for any cognitive operation related to social-value. The schema may be used as an inverse model to calculate the behavioral plan for obtaining an intended social evaluation (Figure 4C). The activation of the vMPFC and PCC during moral judgment is greater when the situation is more realistic (i.e., relevant to the real-life evaluation of self), such as when the decision is situation-based rather than rule-based (Robertson et al., 2007) or when the potential victim of the decision is humanized by mentalizing manipulation (Majdandzic et al., 2012). In daily life, we are intermittently engaged in such behavioral planning on the basis of the social-value of the self, while it is interrupted during execution of a specific attention-demanding task. This appears to be a plausible explanation for the activation of these areas during the conscious resting state (Gusnard et al., 2001; Raichle et al., 2001). Furthermore, the simulation capacity of the social-value schema (i.e., the making of inferences regarding the intentions and plans related to another’s social behavior) may explain the activation of these regions during social-value judgments about others (Craik et al., 1999; Schmitz et al., 2004; Sugiura et al., 2004; Ochsner et al., 2005; Benoit et al., 2010). Given the general role of the vMPFC and PCC in value-based decision making (Rangel et al., 2008; Rushworth et al., 2011) and their specific roles in highly integrated visuospatial and memory retrieval processes (Wagner et al., 2005; Cavanna and Trimble, 2006), respectively, it seems fair to consider the social-value schema as only a subcomponent of the functioning of this neural system.

### Multi-layer structure and cross-layer dynamics

It is further proposed that the three categories of self, or internal schemata, comprise a hierarchical layered structure in that the maturation of one layer, or schema, serves as the basis for the development of the next layer. Additionally, the prediction error generated in one layer may result in an updating of the schema not only in that layer but also in adjacent layers. These cross-layer dynamics may be, in part, responsible for both the integrity of the categories and the ambiguity across the three self-concepts.

The self-layers are assumed to develop in the following order: sensorimotor, interpersonal, and social-value. The development of a higher layer is dependent on the maturation of the internal schema in a lower layer; here, the maturation of the schema denotes the acquisition of the potential to simulate the schema of others.

In terms of the sensorimotor schema, the acquisition of the potential to infer the intention or action goal of others corresponds to an infant’s discovery of an “other” or an agent who has a similar mental mechanism to the self. This discovery of an other is the very basis of the development of the interpersonal schema that requires the execution of social action toward the other and the understanding of the other’s social response. In fact, a similar concept to this simulation potential has been conceptualized in a recent hierarchical self-model as the Bodily Social Self (BSS), which links Phenomenal Self and Narrative Self (Farmer and Tsakiris, 2012); the former seems to correspond to self-body-dedicated (premature) physical self, and the latter to interpersonal self and social-value of self, together, in the proposed model. This simulation capacity, or the BSS, is included in the physical self in this neural-representation model because both types of self are accommodated by the sensorimotor schema. The maturation process, the acquisition of a simulation capacity by the sensorimotor schema, is assumed to develop in the first 6 months of life in infants (Kaye and Fogel, 1980; Cohn and Tronick, 1988), and its failure to develop has been proposed as responsible for the impaired development of sociality in autism (Gergely, 2001).

The next step of development is triggered by the maturation of the interpersonal schema. The acquisition of a simulation capacity, ToM or mentalizing ability, by the interpersonal schema enables one to conceive of the representation of self in another’s mind. The collection of such self-representations in many others’ minds allows abstraction of the value of self to construct the social-value schema. This internalization process is taken for granted in developmental theories of social self-concepts, with the process, presumably, peaking in adolescence (Cooley, 1902; Mead, 1934; Harter, 1985). Accordingly, self-dominant activation during judgment about significant social attributes is observed in the vMPFC in adults and in the dMPFC in adolescents (Pfeifer et al., 2007). This probably reflects the ongoing self-value abstraction process in the interpersonal schema.

The cross-layer functioning of the error-based updating of the schema adds tremendous complexity to one’s self-related experiences as well as to the interpretation of neuroimaging findings. For example, prediction error in the sensory-feedback in response to an action by a subject may produces a sense or belief that the action is performed by another person rather than a feeling of strangeness in one’s own action; that is, error-based updates do not influence the sensorimotor schema but the interpersonal schema. Indeed, the experimental manipulation of sensory-feedback during the moving of a hand by a subject or during the manipulation of an agent on a computer monitor activates the TPJ/pSTS and dMPFC (McGuire et al., 1996a; Farrer and Frith, 2002; Farrer et al., 2003, 2008; Hashimoto and Sakai, 2003; Leube et al., 2003; Fu et al., 2006; Spengler et al., 2009; Yomogida et al., 2010), which are implicated in the interpersonal schema. An abnormal functioning of this cross-layer error-attribution (i.e., attribution to other) is considered to explain several symptoms of schizophrenia, including the delusion of control (Frith et al., 2000; Frith, 2005).

Another example is the case in which prediction error in the self-value layer influences the interpersonal schema. Unexpectedly high or low evaluation of the self by another is assumed to cause change in self-value but, alternatively, may be attributed to an idiosyncratic viewpoint or attitude of the evaluator (interpersonal schema). One example may be a case in which one thinks “his recognizing me as stupid is not because I am stupid, but because he is stupid.” In the experimental setting, feedback evaluation toward the self is typically provided by a small number of alleged evaluators, and it is highly likely that cross-layer attribution does occur. This consideration is congruent with data identifying neural responses that are positively correlated with self-value prediction error (i.e., discrepancy between a subject’s own evaluation of the self and the evaluation by others) in the major components of the interpersonal schema (Korn et al., 2012).

The implementation of hierarchical layer structure and cross-layers error-attribution, in addition to the association-based generation of the internal schema and its prediction-error-based update *per se*, makes this model conform to the Hierarchical Bayesian model based on the free-energy principle (Friston, 2010). This conformity may suggest a future potential sophistication of this model in the Bayesian framework. The proposed model may, therefore, provide an example of successful adoption of this comprehensive framework to cognitive processes that cover perception to higher-level cognition accompanying empirical data.

Several cortical areas may play a unique role in the coordination of functioning across multiple layers. Specifically, the right lateral prefrontal cortex may have a role in resolving conflicts in different layers. This region is activated during sensory-feedback manipulation when it obviously conflicts with motor control (Fink et al., 1999) or when agency-attribution judgment (i.e., self or other) is required (David et al., 2007; Schnell et al., 2007; Farrer et al., 2008). It has been proposed that this region is responsible for an impaired belief-validation process during the mirrored-self misidentification (mirror sign) due to a failure to resolve the conflict between self-face recognition and contingency detection when either process is abnormal (Coltheart, 2007, 2010). Apparently consistent with this view, activation in the right lateral prefrontal cortex is frequently reported during the recognition of self-face or self-body in non-contingent images (Platek et al., 2004, 2006; Sugiura et al., 2005a, 2006, 2008, 2012; Uddin et al., 2005; Devue et al., 2007; Kaplan et al., 2008). Moreover, this region responds to behavior that violates social norms (i.e., error in the interpersonal layer) (Wakusawa et al., 2009) or when there are discrepancies between a subject’s self-evaluation and the evaluation by others (i.e., error in the social-value layer) (Korn et al., 2012).

In summary, the concept of an internal schema with three layers operating under the assumption of cross-layer dynamics provides a relatively simple integrated conceptual framework for the self-concept. In this framework, associative learning and the hierarchical structure of the cortical network appear sufficient to explain the wide range of behavioral, developmental, and neuroimaging findings related to self-cognition.

## Role of Midline Structures

In the proposed model, a majority of the association cortices in both lateral and medial structures critically contribute to some aspect of self. This view may be inconsistent with the notion of a special role for the midline regions in self-cognition, which might be implied by this topic. Based on the size of areas included, however, it is possible to characterize the contribution of the midline regions in the following manner: they are most relevant to the social-value layer, less relevant to the interpersonal layer, and least relevant to the sensorimotor layer. This characterization is largely congruent with the view that has previously been discussed (Uddin et al., 2007).

Within the proposed conceptual framework, the primary interest regarding midline structures concerns the functional demarcation of the neural correlates of each schema. The border of the cortical correlates for each self-layer parallels the functional segregation of the midline structures.

In the frontal lobe, the border between the cortical correlates of the sensorimotor and interpersonal schema may be reasonably defined as the border between the premotor cortex (Brodmann area 6) and the posterior part of the prefrontal cortex (Brodmann area 8) given the motor-associated and amodal nature of these schemata, respectively. A review of neuroimaging findings evaluating the dorsal part of the medial frontal lobe suggests that the border is a few centimeters rostral to the vertical plane crossing through the anterior commissure (AC). Clusters of activation peaks related to attention to action or to sensation are located posteriorly, and those related to concepts are located anteriorly (Seitz et al., 2006). The location of this functional border appears largely congruent with the cytoarchitectonic border between area 6 and area 8 (Geyer, 2004). The border between the regions for the interpersonal and social-value schema, on the other hand, has been defined only functionally. Previous reviews have consistently demonstrated a functional inhomogeneity of the MPFC as the dorsal and ventral regions tend to be involved in cognitive and emotional processes, respectively. However, the proposed level of a horizontal plane for that border has varied across studies (i.e., from running through the AC to 20 mm above the AC) (Amodio and Frith, 2006; Van Overwalle, 2009).

In the medial parietal lobe, the extent of the cortical correlates of the social-value schema remains inconclusive but may encompass the entire precuneus and PCC. This region contains multiple functional subareas that are specialized for the processing of particular components of the implicated roles in this region, such as episodic-memory retrieval, visual imagery, and the representation of a personally familiar place (Sugiura et al., 2005b; Wagner et al., 2005; Cavanna and Trimble, 2006; Summerfield et al., 2009; Zhang and Li, 2012). Although all of these processes appear to be relevant in some way to the processing of contextual roles, a detailed account of the association of these processes with respect to the concept of the social-value schema is extremely premature.

## Conclusion

The framework proposed herein is an attempt to rescue the integrated construct of self from the pessimistic view arguing against the existence of self-specific neural system. The concepts of self appear to be parsimoniously arranged into three categories according to the contexts of awareness and development as well as the implicated cortical regions. According to the proposed model, the internal schema, which represents the learned associations between behavioral output and feedback input, enables the system to engage in forward prediction and explains the sense of self in all three categories. Importantly, the internal schema is not exclusively dedicated to self-cognition but is the very basis of the cognitive system underpinning interaction with a physical or social environment. Additionally, the schemata for these three categories of self comprise a hierarchical layer structure in terms of their developmental and updating processes.

The sensorimotor schema, namely, the association of a motor plan with feedback sensory input acquired through exploratory motor activity, is supported by sensory and motor association cortices and results in a sense of a physical self representing self-agency of action, body-ownership, and bodily self-recognition. As the schema matures, it becomes capable of simulating the intention or action goals of others. This allows one to explore and experience social interaction in which the interpersonal schema, or the association of one’s own social action with subsequent social responses that serve as feedback, is developed in the amodal association cortices of the dMPFC and lateral posterior cortices (e.g., pSTS/TPJ and ATC). While allowing for the experience of the interpersonal self, which is the awareness of self-directed attention or the intention of others, the interpersonal schema also matures to accommodate the representation of the self in another’s mind. The collection of such self-representations in many others’ minds enables the development of the social-value schema, which evaluates one’s social behavior and feedback evaluation. This schema, supported by the vMPFC and PCC, enables the operation of the social self and represents the self as a collection of context-dependent social-values.

The model proposed herein explains the large variety of activated regions that have been reported by studies addressing self-related cognitive processes as well as their involvement in non-self-related processes. It also provides a unique perspective on the relationship between self-cognition and the cognitive system involved in one’s interaction with the physical or social environment. In particular, the assumed layer structure provides for the development, complexity, and integrity of three categories of the self. This view understands the different, but not mutually exclusive, roles of the midline and lateral cortical regions in self-cognition in terms of the different medial–lateral distribution of the three internal schemas. With respect to midline structures, due to the different sizes of the areas that each internal schema occupies, the characteristics of the self may be ranked as follows in term of prominence: social-value self, interpersonal self, and physical self.

## Conflict of Interest Statement

The author declares that the research was conducted in the absence of any commercial or financial relationships that could be construed as a potential conflict of interest.
